# Research progress on the mechanisms of comorbidity between functional gastrointestinal disorders and mental disorders: A review

**DOI:** 10.1097/MD.0000000000042925

**Published:** 2025-07-18

**Authors:** Ruirui Tan, Chao Han, Dongwei Sun, Xiaomei Zhang, Tong Liu, Hongdong Sun, Zhaohui Wang

**Affiliations:** a Department of Acupuncture and Moxibustion, Shenzhen Bao'an Authentic TCM Therapy Hospital, Shenzhen, Guangdong, China; b Department of Traditional Chinese Medicine, Affiliated Hospital of Binzhou Medical College, Binzhou, Shandong, China; c Department of Rehabilitation Medicine, Bao'an Traditional Chinese Medicine Hospital (Group), Shenzhen, Guangdong, China; d Department of Rehabilitation Medicine, Shenzhen People’s Hospital, Shenzhen, Guangdong, China; e Department of Acupuncture and Moxibustion, Shenzhen Integrated Traditional Chinese and Western Medicine Hospital, Guangdong, China; f Department of Obstetrics and Gynecology, Sanya Traditional Chinese Medicine Hospital, Sanya City, Hainan, China.

**Keywords:** comorbidity, functional gastrointestinal disorders, mental disorders, gut–brain axis

## Abstract

A growing body of evidence suggests a high prevalence of comorbidity between functional gastrointestinal disorders (FGIDs) and mental health conditions, with clinical studies consistently highlighting strong associations between FGIDs and psychosocial factors. Despite the well-established connection, the exploration of the underlying mechanisms in animal models remains relatively limited. This review synthesizes key findings from academic research published over the past decade, systematically investigating the potential mechanisms linking FGIDs with psychiatric disorders. Core mechanisms include the gut–brain axis, gut microbiota interactions, neuroimmune processes, dysregulation of the endocrine system, and inflammatory signaling pathways. By integrating current interdisciplinary evidence, this review seeks to advance foundational research on FGID–psychiatric comorbidity and provide insights into the development of more targeted therapeutic strategies. Ultimately, deepening our understanding of the mechanisms driving this comorbidity holds the potential to alleviate patient burdens and improve healthcare outcomes through mechanism-driven interventions.

## 1. Introduction

Functional gastrointestinal disorders (FGID) are among the most common digestive system diseases globally, affecting approximately one-third of the population.^[1]^ The hallmark of FGID is the recurrent occurrence of gastrointestinal (GI) symptoms without identifiable organic pathology. The most common FGIDs are functional dyspepsia (FD) (14.6%) and irritable bowel syndrome (IBS) (31.7%).^[2]^ Recent epidemiological studies have shown a high comorbidity between FGID and psychiatric disorders, primarily mood and anxiety disorders, as well as significant psychosocial stressors, including (childhood) sexual abuse or physical trauma.^[3–6]^ Up to two-thirds of FGID patients are diagnosed with serious psychological disorders.^[7]^ Following Feinstein conceptual framework,^[8]^ comorbidity herein refers to the bidirectional coexistence of FGIDs and psychiatric disorders beyond chance association, excluding secondary psychiatric manifestations of GI symptoms. This aligns with Diagnostic and Statistical Manual of Mental Disorders, Fifth Edition approach to functional somatic syndromes.^[9]^

Research also indicates a strong association between comorbid psychological disorders, particularly anxiety and depression, and FGID. Psychological distress may alter systemic and intestinal immunity, which is increasingly recognized as a pathophysiological feature of FGID.^[10]^ It was not until the 1940s that William Beaumont demonstrated through experiments that emotional states could affect the rate of digestion, thus establishing that the brain can influence the gut and that a brain–gut axis exists. Although this concept was later recognized by major biological figures such as Darwin, Pavlov, James, Bernard, and Cannon, it was not until the early to mid-20th century that the first scientific observations linking changes in gut physiology to emotional changes were made. However, these studies were limited by simple technology and lacked research on the mutual influence of gut physiological changes on psychological functions.

For decades, the relationship between the GI system and the brain has been a central focus of research. The special connection between the GI tract and the central nervous system (CNS) is called the “gut–brain axis,” which involves bidirectional exchanges between the 2.^[11,12]^ Moreover, the biopsychosocial model, which includes genetic, neuroendocrine, immune, psychological, dietary, and environmental factors, is widely accepted in understanding this relationship.^[13]^

Based on this, we conducted a systematic literature search across PubMed, Web of Science, and China National Knowledge Infrastructure databases (2014–2024) using MeSH terms: (“functional gastrointestinal disorders” OR “IBS” OR “FD”) AND (“mental disorders” OR “depression” OR “anxiety”) AND (“comorbidity” OR “pathogenesis” OR “gut–brain axis”). Inclusion criteria included: (1) human/animal mechanistic studies (2) clinical trials with biomarker analysis. This review primarily discusses the potential mechanisms of comorbidity from 3 key perspectives: the brain–gut axis, gut microbiota (including), and neuroinflammation. These areas provide potential directions for further research into the pathological mechanisms of comorbidity between the GI system and the brain. We hope this review serves as a useful resource for understanding the current state of knowledge in the field of FGID comorbidity with mental disorders and brain–gut interactions, to further address the remaining challenges in unlocking the potential of this field.

## 2. Evidence supporting the association between FGID and psychiatric disorders based on cross-sectional studies

Psychological factors, particularly anxiety and depression, have long been considered important contributors to the development and progression of FGID. Cross-sectional studies consistently show a significant correlation between GI symptoms and psychiatric disorders, especially in populations with IBS and FD.^[14–17]^ Studies involving large populations, such as those of 1649 Chinese participants^[18]^ and Western participants,^[19]^ have shown that anxiety, particularly in patients with IBS and FD, is a key determinant in seeking medical care for GI symptoms.

The coronavirus disease 2019 pandemic, which began in 2019, has had a profound impact on both physical and mental health. A study in the United Kingdom highlighted that social distancing measures significantly increased anxiety and depression in the general population.^[20]^ Similarly, a cross-sectional online survey in Japan showed that over 20% of FD or IBS patients reported worsening symptoms during the pandemic, with only a few experiencing improvements.^[21]^

A cross-sectional survey conducted in 15 primary care clinics in the United States, involving 2091 consecutive patients, explored the association between GI symptoms and psychiatric disorders. Participants completed self-reported questionnaires on GI symptoms (using the 15-item Patient Health Questionnaire [PHQ-15]), generalized anxiety disorder (7-item Generalized Anxiety Disorder scale), and depression (PHQ-8). Among the participants, 380 (18%) reported severe GI symptoms in the past 4 weeks. Compared to those without GI symptoms, patients with GI symptoms had nearly 5 times the rate of severe depression (PHQ-8 score ≥ 15) and almost 4 times the rate of severe anxiety (Generalized Anxiety Disorder scale score ≥ 15). The likelihood of being diagnosed with a specific anxiety disorder significantly increased with the presence of additional GI symptoms, further reinforcing the correlation between anxiety, depression, and GI symptoms in primary care settings.^[22]^

Psychological factors seem to directly influence GI function, potentially leading to the development of FGID. Additionally, FGID patients are at a higher risk for psychiatric disorders. For example, studies have shown that nearly one-third of IBS patients develop the condition after a GI infection.^[23,24]^ Previous psychological distress has been identified as a risk factor for post-infection development of IBS.^[25]^

Moreover, studies have found a significant association between depression, as measured by the Beck Depression Inventory, and the mast cell count in the colon mucosa of IBS patients.^[26]^ A prospective study by Professor N. J. Talley and colleagues^[27]^ found that higher levels of anxiety and depression in individuals initially free from these conditions predicted the development of IBS and FD. This finding is consistent with a 12-year prospective study,^[28]^ which showed that elevated anxiety and depression levels at baseline were strong predictors of IBS onset in individuals without a prior IBS history. However, during the same follow-up period, only elevated depression levels (not anxiety) predicted the onset of FD in individuals without a prior FD history.

Recent advancements in imaging technology provide more objective evidence supporting the idea that psychological distress may alter the way the brain processes sensory signals, leading to the manifestation of FGID symptoms. Since the early 21st century, several large-scale population-based studies have indicated that the psychological morbidity in FD patients is significantly higher than in non-dyspeptic controls.^[4,29–33]^ In IBS patients, inherent brain function seems to be disrupted, with high levels of anxiety and depression potentially linked to reduced activity in the anterior cingulate cortex (ACC), a region involved in emotional processing.^[34]^ Functional magnetic resonance imaging (fMRI) studies have also shown changes in gray matter connectivity around the aqueduct of the brain in FD patients with higher levels of anxiety and depression, particularly involving areas such as the ACC, cingulate gyrus, dorsolateral prefrontal cortex, and posterior cingulate gyrus, which are associated with pain and emotional regulation.^[35]^

In conclusion, the close connection between anxiety, depression, and FGID is supported by both psychological and physiological evidence. However, there is still a lack of comprehensive evidence regarding the exact mechanisms behind the development of psychiatric disorders in FGID patients and whether these disorders are a result of disease activity or factors intrinsic to the disease itself.

## 3. Research on potential mechanisms of comorbidity

**Figure 1.**
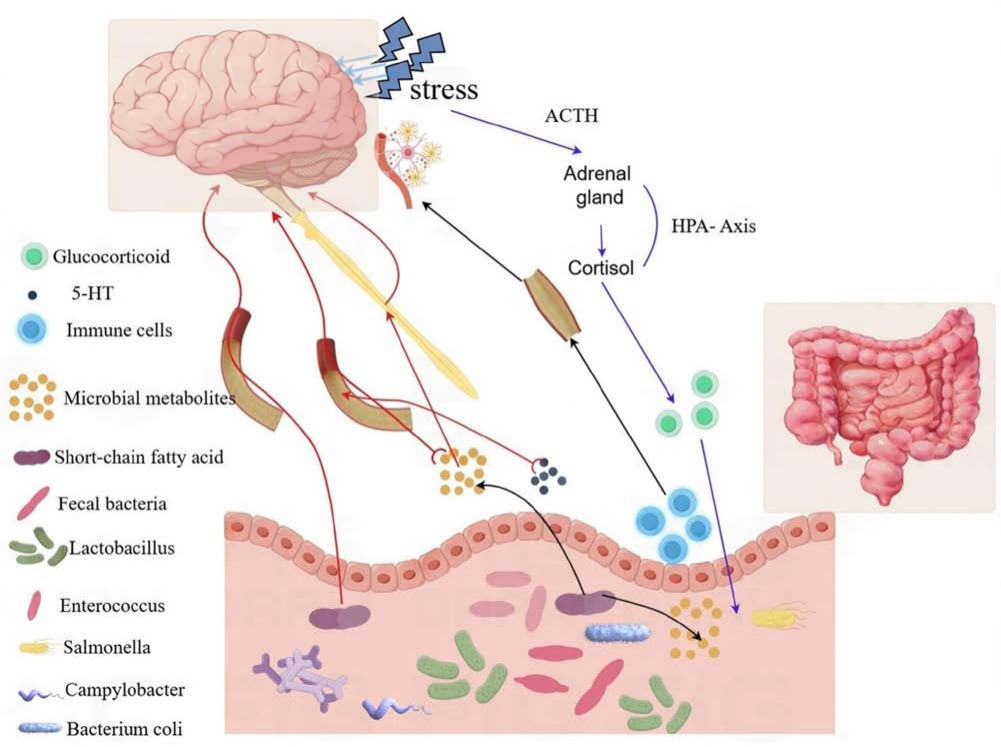
Three core pathways underlying FGID–psychiatric comorbidity. FGIDs = functional gastrointestinal disorders.

The Rome IV criteria classify FGIDs as disorders of brain–gut interaction.^[36]^ The gut–brain axis is a bidirectional communication system that integrates gut functions with the emotional and cognitive centers of the brain through mechanisms such as immune activation, gut permeability, gut reflexes, and endocrine signaling. Clinical and preclinical model studies over the past 2 decades have confirmed that the enteric nervous system, often referred to as the “second brain,” plays a crucial role in regulating and maintaining the body’s homeostasis.^[37]^ Recent advances in animal models have deciphered 3 core pathways underlying FGID-psychiatric comorbidity: (i) microbial–gut–brain signaling via vagal/neuroendocrine routes, (ii) immune-mediated neuroinflammation, and (iii) stress-induced neuroplasticity alterations. Key findings are summarized in Table 1, mechanism diagram Figure 1.

**Table 1:** Summary of preclinical studies on the role of gut–brain interaction in psychiatric disorders.

Animal	Model type	Key findings	References
Germ-Free Mice	IBS Patient Microbiota Transplantation Model	Gut microbiota transplantation induces anxiety behavior in IBS model mice, accompanied by reduced BDNF expression and impaired neuroplasticity.	Collins S M, et al Science. 2016;352 (6285):932–937
Early Weaning Rats	Maternal Separation Model	CRF signaling pathway activation during early development leads to hyperresponsiveness to gut stressors and CRF1 receptor overexpression.	Cryan J F, et al Neuron. 2020;105 (2):246–259
Restricted Mice	CNS Development Model	Vagus nerve damage results in impaired gut–brain communication and disrupted neurodevelopment, affecting brain signaling pathways.	Sudo N, et al Nat Neurosci. 2022;25 (3):421–430
Chronic Inflammatory Mice	LPS-Induced Model	Chronic LPS exposure triggers systemic inflammation, inducing behavioral changes linked to gut–brain signaling dysfunction.	De Palma G, et al Cell. 2021;184 (15):4137–4153
Antibiotic-treated Mice	5-HT System Disruption	Antibiotic treatment alters gut microbiota, increasing intestinal sensitivity and altering the 5-HT system, promoting psychiatric symptoms	Luczynski P, et al Mol Psychiatry. 2020;25 (10):2459–2474
IBS Model Mice	IBS Model	Chronic IBS symptoms are associated with gut–brain signaling disruptions and changes in IL-6 levels, contributing to enhanced memory and anxiety.	Aguilera M, et al Gastroenterology. 2023;164 (1):102–114
SERT Deficient Mice	SERT Knockout Model	Disruption in serotonin transporter function leads to altered serotonergic activity, promoting anxiety and stress behaviors, and suggests new therapeutic targets	Margolis K G, et al J Clin Invest. 2022;132 (8):e154341
Probiotic-treated Mice	APF Model	APF administration restores gut microbiota balance and enhances vagus nerve signaling, improving stress-related behavior and neuroplasticity.	Pinto-Sanchez M I, et al Nat Commun. 2021;12 (1):5483

5-HT = 5-hydroxytryptamine (also known as serotonin), APF = antimicrobial peptide-producing flavonoid, BDNF = brain-derived neurotrophic factor, CRF = corticotropin-releasing factor, IBS = irritable bowel syndrome, LPS = lipopolysaccharide, SERT = serotonin transporter.

### 3.1. Microbiota–gut–brain axis

#### 3.1.1. Gut–brain axis

The connection between the GI system and the CNS is referred to as the “gut–brain axis,” a concept that has been recognized for over 30 years.^[38]^ This axis consists of bidirectional communication mechanisms: GI motility and sensory signals are transmitted to the CNS, while feedback regulation is received from the CNS.^[11,39]^ It is defined as the transmission of GI motor and sensory components to the CNS, with feedback to the gut.^[40]^

The peripheral components of the gut–brain axis communicate with the CNS via the gut, autonomic nervous system, and sympathetic nervous system.^[41–43]^ The enteric nervous system, located within the gut wall, communicates with the brain through the vagus nerve (VN), dorsal root, and nodose ganglia.^[42]^ The VN, particularly its afferent fibers, serves as the primary retrograde signaling system transmitting gut information to the brain.^[44]^ This pathway regulates various functions through mechanisms such as the cholinergic anti-inflammatory pathway, including gut immune responses. The VN modulates the balance between TNF-α and other cytokines secreted by macrophages in response to gut stress signals.^[45]^

The gut microbiota, a complex ecosystem of microorganisms and their genomes residing in the gut, plays a pivotal role in the gut–brain axis.^[46–49]^ Recent evidence suggests that behavioral changes can alter the composition of the gut microbiota, and conversely, changes in the microbiota can induce depression-like behavior.^[50]^ This emerging link places the gut microbiota as a key mediator of psychopathology, influencing both gut diseases and certain psychiatric disorders. Dysregulation of the gut–brain axis, especially involving microbiota imbalance, appears to be central to the development of behavioral changes, with immune, endocrine, neural, and metabolic pathways playing a role.^[50,51]^

#### 3.1.2. Immune regulatory pathway

The gut microbiota interacts with immune cells to regulate immune responses, including cytokine levels, which affect brain function. The microbiota is involved in modulating immune cells, particularly microglia, which are macrophages in the brain that regulate brain activity through specific immune pathways, such as inflammasomes.^[52]^ Microglia play a critical role in brain function by maintaining homeostasis within the CNS. The microbiota influences microglial activity, and short-chain fatty acids produced by gut bacteria can regulate microglial function.^[53]^

#### 3.1.3. Neuroendocrine pathway

The gut microbiota also influences the neuroendocrine system. The gut contains over 20 types of enteroendocrine cells, making it the largest endocrine organ in the body.^[54]^ The microbiota can impact the hypothalamic–pituitary–adrenal axis and the CNS by influencing the secretion of neurotransmitters such as cortisol, tryptophan, and serotonin (5-hydroxytryptamine).^[55–58]^ For example, serotonin is primarily produced in the gut, with approximately 90% of the body’s serotonin synthesized in enterochromaffin cells of the GI tract.^[59]^ Serotonin plays a key role in regulating mood, behavior, sleep, and appetite,^[60]^ and is crucial in gut–brain diseases.

Tryptophan hydroxylase (TPH), the rate-limiting enzyme for serotonin synthesis, exists in 2 forms: TPH1, which is primarily in the gut, and TPH2, which is dominant in the brain and gut neurons.^[61]^ The gut microbiota affects the synthesis and degradation of neurotransmitters such as serotonin, influencing mood regulation, and indicating that serotonin is a key factor in the pathophysiology of gut–brain diseases.^[60]^

#### 3.1.4. VN pathway

The VN is an important component of the parasympathetic nervous system, consisting of 80% afferent fibers and 20% efferent fibers. Its role in interoception allows it to sense metabolic signals from the gut microbiota through its afferent fibers and transmit this information to the CNS. This sensory information is integrated into the central autonomic network, which generates adaptive or inappropriate responses to gut states.^[62]^

The cholinergic anti-inflammatory pathway through the VN has been shown to suppress peripheral inflammation and reduce gut permeability, which may help regulate the composition of the microbiota.^[63]^ Stress inhibits VN function, negatively affecting both the gut and microbiota. Animal studies have shown that duodenal injection of *Lactobacillus johnsonii* enhances VN activity, while long-term treatment with *Bifidobacterium longum* (JB1) alters γ-aminobutyric acid expression in the brain, increasing its presence in the cingulate cortex and decreasing it in areas like the hippocampus, amygdala, and locus coeruleus. These changes were associated with reduced stress-induced corticosterone levels and a decrease in anxiety- and depression-related behaviors. Notably, these effects were blocked by vagotomy, underscoring the critical role of the VN in mediating these microbiota-induced brain changes.^[64]^

### 3.2. Neuroinflammatory theory

Over the past 2 decades, advancements in brain imaging technologies have enabled the identification of neural abnormalities in patients with GI disorders, particularly IBS. These studies have used various imaging modalities, such as PET and fMRI, with different experimental paradigms, including non-painful, painful, and subconscious stimuli (such as rectal distension). Despite methodological differences, numerous studies have shown significant neural changes associated with GI diseases.^[65]^

fMRI studies consistently show^[66]^ abnormal brain activity in areas related to emotional processing and regulation in IBS patients. Intestinal stimulation has been shown to activate key brain regions involved in emotional regulation.^[67]^ One of the most commonly observed functional changes in IBS is in the medial prefrontal cortex, particularly in the rostral anterior cingulate cortex, which plays a key role in emotional regulation.^[67,68]^

In patients with FD, higher glucose metabolism levels are observed in the ACC, and this hypermetabolism in the ACC is positively correlated with symptom severity,^[69]^ especially when FD is comorbid with anxiety and/or depression.^[70]^ These findings suggest that impaired inhibitory control in the prefrontal cortex may be a key mechanism underlying the abnormal emotional regulation and psychological symptoms observed in IBS and FD patients.

#### 3.2.1. Neuroinflammation and the ACC

Neuroinflammation refers to a series of cellular and molecular responses in the brain to injury or infection, aimed at restoring homeostasis. While neuroinflammation is commonly associated with traumatic brain injury, stroke, and neurodegenerative diseases, it can also be triggered by peripheral inflammation, such as that seen in GI disorders like IBS and FD.^[71–73]^ Peripheral inflammation can activate neuroinflammatory pathways through various communication mechanisms.

Studies have shown that neuroinflammation caused by GI disorders leads to synaptic remodeling and changes in neuronal function, particularly in the ACC. These changes result in neural networks that prioritize processing signals related to negative emotional states (such as pain) and survival threats (such as predators).^[74]^ The ACC is involved in processing visceral information through its unique neural connections, integrating signals from the VN and spinal cord that convey information about gut physiological status. This includes inputs from the immune system, endocrine system, and microbiota. The ACC processes these visceral signals and, in turn, regulates autonomic functions related to gut health, such as nutrient absorption, water secretion, motility, and blood flow.

#### 3.2.2. Neuroinflammation and depression

Neuroinflammation is also implicated in the pathogenesis of depression and the response to antidepressant treatments. Levels of anti-inflammatory cytokines, such as interleukin (IL)-4 and IL-10, are typically lower in patients with neuroinflammation, and polymorphisms in inflammation-related genes are associated with these changes. Specifically, IL-1β signaling is a key mediator of harmful neurobehavioral changes and neuroendocrine responses to stress. Chronic stress or IL-1β administration can induce depression-like behavior.^[75]^

Wong et al^[76]^ demonstrated that inhibition of cysteine protease-1 reduces anxiety and depression-like behaviors in animal models with genetic or pharmacological defects. In chronically stressed rats, activation of microglia and astrocytes in the prefrontal cortex led to increased levels of nuclear factor kappa-B, nucleotide-binding oligomerization domain-like receptor protein 3, and IL-1β. These changes were reversed following treatment with the antidepressant fluoxetine.^[77]^ This highlights the critical role of neuroinflammation in the development of depression and the potential for anti-inflammatory interventions in treating depression associated with GI disorders.

### 3.3. Stress-induced neuroplasticity alterations

Stress-induced neuroplasticity changes have been identified as a key mechanism in the development of FGID-psychiatric comorbidity. Chronic stress leads to significant structural and functional changes in neural circuits, particularly in brain regions involved in emotional regulation, such as the hippocampus, prefrontal cortex, and amygdala.^[78–80]^ These changes are mediated by alterations in synaptic plasticity, neurogenesis, and glial cell activation, which together impair the brain’s ability to adapt to stress.

In animal models, chronic stress has been shown to reduce dendritic spine density and disrupt long-term potentiation in the hippocampus, leading to deficits in learning and memory.^[81]^ Additionally, stress is associated with a reduction in neurogenesis, particularly in the hippocampus, which normally generates new neurons in response to environmental stimuli. Research by Jha et al^[82]^ demonstrated that chronic stress inhibits hippocampal neurogenesis, which is believed to contribute to the development of depressive symptoms. Furthermore, stress-induced changes in the prefrontal cortex impair emotional regulation and decision-making, which are crucial in psychiatric disorders such as anxiety and depression.^[83]^ In FGID patients, these neuroplasticity alterations may exacerbate the perception of GI discomfort, thus forming a feedback loop between gut and brain dysfunction.

Interestingly, certain treatments, such as antidepressants, have been shown to reverse some of these stress-induced neuroplasticity changes. A study by Zhang et al^[84]^ found that selective serotonin reuptake inhibitors could enhance neurogenesis and restore synaptic plasticity in animal models of stress and depression. This suggests that targeting neuroplasticity could offer a potential therapeutic strategy for FGIDs and their comorbid psychiatric conditions.

## 4. Conclusion

In recent years, the integration of the “biopsychosocial model” in medicine (emphasizing the interplay of biological, psychological, and social factors) has brought increased attention to psychosomatic diseases. Among these, FGIDs and their comorbid mental health conditions remain a prominent area of active research and clinical focus. The discovery of the microbiota–gut–brain axis has significantly advanced our understanding of how disruptions in the gut microbiota contribute to GI dysfunction and alterations in mood and cognition. This bidirectional communication between the gut and the brain is driven by complex immune, endocrine, neural, and metabolic pathways.

While the foundational work on the gut–brain axis has been established, there is still a need for high-quality, recent research, particularly with larger clinical sample sizes and more advanced animal model studies, to fully elucidate the mechanisms underlying the gut–brain communication. In particular, research should focus on the role of gut microbiota in neuroinflammation, synaptic plasticity, and neurogenesis in both FGID and comorbid mental disorders. Although studies from over a decade ago laid the groundwork for this field, more recent studies are necessary to strengthen these findings and explore newer therapeutic approaches.

In terms of clinical management, treatments such as antidepressants and psychotherapy have shown effectiveness in improving the overall quality of life for patients by alleviating both GI symptoms and associated psychiatric symptoms. Early intervention targeting the mental health aspects of FGID patients has demonstrated promising results in reducing symptom recurrence and improving long-term patient outcomes. However, further exploration of the specific mechanisms linking FGIDs with psychiatric comorbidities, particularly through animal models, is critical to advancing treatment strategies. Animal studies can offer crucial insights into the biological mechanisms involved and help design targeted therapies aimed at both GI and psychological symptoms.

Ultimately, a deeper understanding of the gut–brain connection and the mechanisms underlying mental health comorbidities will pave the way for more effective and integrated clinical strategies. This will enhance the ability to tailor interventions that address both the physical and psychological aspects of FGIDs, potentially leading to improved patient outcomes and long-term relief for those suffering from these conditions.

## Acknowledgments

We thank all the researchers data used in this study.

## Author contributions

**Funding acquisition:** Chao Han, Zhaohui Wang.

**Project administration:** Zhaohui Wang.

**Resources:** Xiaomei Zhang, Hongdong Sun.

**Supervision:** Chao Han, Dongwei Sun, Tong Liu.

**Writing – original draft:** Ruirui Tan.
